# Neural spiking for causal inference and learning

**DOI:** 10.1371/journal.pcbi.1011005

**Published:** 2023-04-04

**Authors:** Benjamin James Lansdell, Konrad Paul Kording

**Affiliations:** 1 Department of Bioengineering, University of Pennsylvania, Philadelphia, Pennsylvania, United States of America; 2 Department of Neuroscience, University of Pennsylvania, Philadelphia, Pennsylvania, United States of America; Durham University, UNITED KINGDOM

## Abstract

When a neuron is driven beyond its threshold, it spikes. The fact that it does not communicate its continuous membrane potential is usually seen as a computational liability. Here we show that this spiking mechanism allows neurons to produce an unbiased estimate of their causal influence, and a way of approximating gradient descent-based learning. Importantly, neither activity of upstream neurons, which act as confounders, nor downstream non-linearities bias the results. We show how spiking enables neurons to solve causal estimation problems and that local plasticity can approximate gradient descent using spike discontinuity learning.

## Author summary

Despite significant research, models of spiking neural networks still lag behind artificial neural networks in terms of performance in machine learning and modeling cognitive tasks. Given this, we may wonder, why do neurons spike? A key problem that must be solved in any neural network is the credit assignment problem. That is, how does a neuron know its effect on downstream computation and rewards, and thus how it should change its synaptic weights to improve? Artificial neural networks solve this problem with the back-propagation algorithm. We are still seeking to understand how biological neural networks effectively solve this problem. In this work we show that the discontinuous, all-or-none spiking response of a neuron can in fact be used to estimate a neuron’s causal effect on downstream processes. Inspired by methods from econometrics, we show that the thresholded response of a neuron can be used to get at that neuron’s unique contribution to a reward signal, separating it from other neurons whose activity it may be correlated with. This proposal provides insights into a novel function of spiking that we explore in simple networks and learning tasks.

## Introduction

Most nervous systems communicate and process information utilizing spiking. Yet machine learning mostly uses artificial neural networks with continuous activities. Computationally, despite a lot of recent progress [1–5], it remains challenging to create spiking neural networks that perform comparably to continuous artificial networks. Instead, spiking is generally seen as a disadvantage—it is difficult to propagate gradients through a discontinuity, and thus to train spiking networks. This disparity between biological neurons that spike and artificial neurons that are continuous raises the question, what are the computational benefits of spiking?

There are, of course, pragmatic reasons for spiking: spiking may be more energy efficient [6, 7], spiking allows for reliable transmission over long distances [8], and spike timing codes may allow for more transmission bandwidth [9]. Yet, despite these ideas, we may still wonder if there are computational benefits of spikes that balance the apparent disparity in the learning abilities of spiking and artificial networks.

A key computational problem in both biological and artificial settings is the credit assignment problem [10]. When performance is sub-optimal, the brain needs to decide which activities or weights should be different. Credit assignment is fundamentally a causal estimation problem—which neurons are responsible for the bad performance, and not just correlated with bad performance? Solving such problems is difficult because of confounding: if a neuron of interest was active during bad performance it could be that it was responsible, or it could be that another neuron whose activity is correlated with the neuron of interest was responsible. In general, confounding happens if a variable affects both another variable of interest and the performance. Even when a fixed stimulus is presented repeatedly, neurons exhibit complicated correlation structures [11–15] which confounds a neuron’s estimate of its causal effect. This prompts us to ask how neurons can solve causal estimation problems.

The gold-standard approach to causal inference is randomized perturbation. If a neuron occasionally adds an extra spike (or removes one), it could readily estimate its causal effect by correlating the extra spikes with performance. Such perturbations come at a cost, since the noise can degrade performance. This class of learning methods has been extensively explored [16–22]. However, despite important special cases [17, 19, 23], in general it is not clear how a neuron may know its own noise level. Thus we may wonder if neurons estimate their causal effect without random perturbations.

How else could neurons estimate their causal effect? Over a short time window, a neuron either does or does not spike. Comparing the average reward when the neuron spikes versus does not spike gives a confounded estimate of the neuron’s effect. Because neurons are correlated, a given neuron spiking is associated with a different network state than that neuron not-spiking. And it is this difference in network state that may account for an observed difference in reward, not specifically the neuron’s activity. Simple correlations will give wrong causal estimates.

However, the key insight in this paper is that the story is different when comparing the average reward in times when the neuron *barely* spikes versus when it *almost* spikes. The difference in the state of the network in the barely spikes versus almost spikes case is negligible, the only difference is the fact that in one case the neuron spiked and in the other case the neuron did not. Any difference in observed reward can therefore *only* be attributed to the neuron’s activity. In this way the spiking discontinuity may allow neurons to estimate their causal effect.

Here we propose the spiking discontinuity is used by a neuron to efficiently estimate its causal effect. Once a neuron can estimate its causal effect, it can use this knowledge to calculate gradients and adjust its synaptic strengths. We show that this idea suggests learning rules that allows a network of neurons to learn to maximize reward, particularly in the presence of confounded inputs. We demonstrate the rule in simple models. The discontinuity-based method provides a novel and plausible account of how neurons learn their causal effect.

## Results

### Causal inference in neural networks

Though not previously recognized as such, the credit assignment problem is a causal inference problem: how can a neuron know its causal effect on an output and subsequent reward? This section shows how this idea can be made more precise.

Causal effects are formally defined in the context of a certain type of probabilistic graphical model—the causal Bayesian network—while a spiking neural network is a dynamical, stochastic process. Thus before we can understand how a neuron can use its spiking discontinuity to do causal inference we must first understand how a causal effect can be defined for a neural network. We present two results:

- First, we lay out how a neural network can be described as a type of causal Bayesian network (CBN).
- Second, assuming such a CBN, we relate the causal effect of a neuron on a reward function to a finite difference approximation of the gradient of reward with respect to neural activity.

Together these results show how causal inference relates to gradient-based learning, particularly in spiking neural networks.

#### Result I

#### From dynamic neural networks to probabilistic graphical models

We develop this idea in the context of a supervised learning setting. To formalize causal effects in this setting, we thus first have to think about how supervised learning might be performed by a spiking, dynamically integrating network of neurons (see, for example, the solution by Guergiuev et al 2016 [24]). That is, for simplicity, to avoid problems to do with temporal credit assignment, we consider a neural network that receives immediate feedback/reward on the quality of the computation, potentially provided by an internal critic (similar to the setup of [24]). Spiking neural networks generally operate dynamically where activities unfold over time, yet supervised learning in an artificial neural network typically has no explicit dynamics—the state of a neuron is only a function of its current inputs, not its previous inputs.

To accommodate these differences, we consider the following learning problem. Consider a population of *N* neurons. Let *v*_*i*_(*t*) denote the membrane potential of neuron *i* at time *t*, having leaky integrate-and-fire dynamics:

$${\dot{v}}_{i}=-g_{L}v_{i}+\sum w_{ij}\left(x_{j}+\eta_{j}\right),\;v\left(t^{+}\right)=v_{\text{r}},\text{if}\;v\left(t\right)=\theta .$$

for leak term *g*_*L*_, reset voltage *v*_r_ and threshold *θ*. The network is assumed to have a feedforward structure. The neurons receive inputs from an input layer **x**(*t*), along with a noise process *η*_*j*_(*t*), weighted by synaptic weights *w*_*ij*_. The network is presented with this input stimulus for a fixed period of *T* seconds. Let the variable *h*_*i*_(*t*) denote the neuron’s spiking indicator function: *h*_*i*_(*t*) = ∑*δ*(*t* − *t*_*s*_) if neuron *i* spikes at times *t*_*s*_. Post-synaptic current, *s*_*i*_(*t*), is generated according to the dynamics $\tau_{s}\dot{s}=-s_{i}+h_{i}\left(t\right)$. On the basis of the output of the neurons, a reward signal *r* is generated, assumed to be a function of the filtered currents **s**(*t*): *r*(*t*) = *r*(**s**(*t*)). Here we assume that *T* is sufficiently long for the network to have received an input, produced an output, and for feedback to have been distributed to the system (e.g. in the form of dopamine signaling a reward prediction error [25]). Given this network, then, the learning problem is for each neuron to adjust its weights to maximize reward, using an estimate of its causal effect on reward.

As described in the introduction, to apply the spiking discontinuity method to estimate causal effects, we have to track how close a neuron is to spiking. Over the time period *T*, to distinguish between just-above-threshold inputs from well-above-threshold inputs, we also consider the input drive to the neuron: *u*_*i*_(*t*), which is the integrated input to the neuron, except without the reset mechanism.

$${\dot{u}}_{i}=-g_{L}u_{i}+\sum w_{ij}\left(x_{j}+\eta_{j}\right).$$

By tracking integrated inputs with a reset mechanism, then the value *Z*_*i*_ = max_0≤*t*≤*T*_ *u*_*i*_(*t*) tells us if neuron *i* received inputs that placed it well above threshold, or just above threshold.

To compute what the network’s output is for the given input over *T* seconds, we define a set of random variables, **X**, **Z**, **H**, **S**, *R* that aggregate the underlying dynamical (and spiking) variables, **x**(*t*), **z**(*t*), **h**(*t*), **s**(*t*) and *r*(*t*), respectively. Specifically, we define aggregating functionals *f*_[⋅]_ to be summaries of the underlying dynamical variable activities. When the network dynamics are irregular [26], these aggregate variables will be approximately independent across subsequent windows of sufficient duration *T*. The dynamics given by the noisy LIF network generate an ergodic Markov process with a stationary distribution. That is, from a dynamical network, we have a set of random variables Φ that summarize the state of the network and can be considered I.I.D. draws from some distribution, which depends on the network’s weights and other parameters, Θ (e.g. noise magnitude and correlation): Φ ≔ (**X**, **Z**, **H**, **S**, *R*) ∼ *ρ*(⋅; Θ).

The functionals are required to only depend on one underlying dynamical variable. The spiking discontinuity approach requires that *H* is an indicator functional, simply indicating the occurrence of a spike or not within window *T*; it could instead be defined directly in terms of *Z*. The random variable *Z* is required to have the form defined above, a maximum of the integrated drive. The choice of functionals is required to be such that, if there is a dependence between two underlying dynamical variables (e.g. *h* and *x*), then there is also some statistical dependence between these variables in the aggregated variables. I.e. a trivial functional *f*_*R*_(*r*) = 0 would destroy any dependence between *X* and *R*. Given these considerations, for the subsequent analysis, the following choices are used:

$$\begin{array}{cc} X & =f_{X}\left(x\right)=\frac{1}{T}\int_{0}^{T}x\left(t\right)\;dt,\\ Z & =f_{Z}\left(u\right)=\underset{0\leq t\leq T}{\text{max}}\;u_{i}\left(t\right),\\ H_{i} & =f_{H}\left(h_{i}\right)=\{\begin{array}{cc} 1, & \int_{0}^{T}h_{i}\left(t\right)\;dt>0,\\ 0, & \text{otherwise} \end{array}\\ S & =f_{S}\left(s\right)=s\left(T\right)\\ R & =f_{R}\left(r\right)=r\left(s\left(T\right)\right) \end{array}$$

These choices were made since they showed better empirical performance than, e.g. taking the mean over *T* for **S** and *R*. Along with the parameters of the underlying dynamical neural network, these choices determine the form of the distribution *ρ*. The learning problem can now be framed as: how can parameters Θ be adjusted such that $E_{\rho }\left(R\right)$ is maximized? Below we show how the causal effect of a neuron on reward can be defined and used to maximize this reward.

#### A causal Bayesian network of a neural network, and a neuron’s causal effect on reward

What is the causal effect of a neuron on a reward signal? Given the distribution *ρ* over the random variables (**X**, **Z**, **H**, **S**, *R*), we can use the theory of causal Bayesian networks to formalize the causal effect of a neuron’s activity on reward [27].

**Definition 1**. In a causal Bayesian network, the probability distribution *ρ* is factored according to a graph, G=(N,E) [27]. The edges E in the graph represent causal relationships between the nodes N; the graph is both directed and acyclic (a DAG). The standard definition of a causal Bayesian model imposes two constraints on the distribution *ρ*, relating to:

1. Conditional Independence: nodes are conditionally independent of their non-descendants given their parents,
  $$\rho \left(Y=y\right)=\prod_{n\in N}^{N}\text{Pr}\left(Y_{n}=y_{n}|{\text{Pa}}_{n}\right),$$
  where Pa_*n*_ represents the parents of node *n*.
2. The Effect of Interventions: when a variable is *intervened* upon, forced to take a given value, the edge between that variable and its parents is severed, changing the data-generating distribution. That is, if we intervene on a node *j*, then the interventional distribution $\rho_{Y_{j}=y_{j}}$ is
  $$\rho_{Y_{j}=y_{j}}\left(Y=y\right)=\delta \left(Y_{j}=y_{j}\right)\prod_{n\neq j}\text{Pr}\left(Y_{n}=y_{n}|{\text{Pa}}_{n}\right).$$
  Where node *j* has been forced to take a value, *y*_*j*_, and the dependence on its parents has been severed.

**Fig 1:**
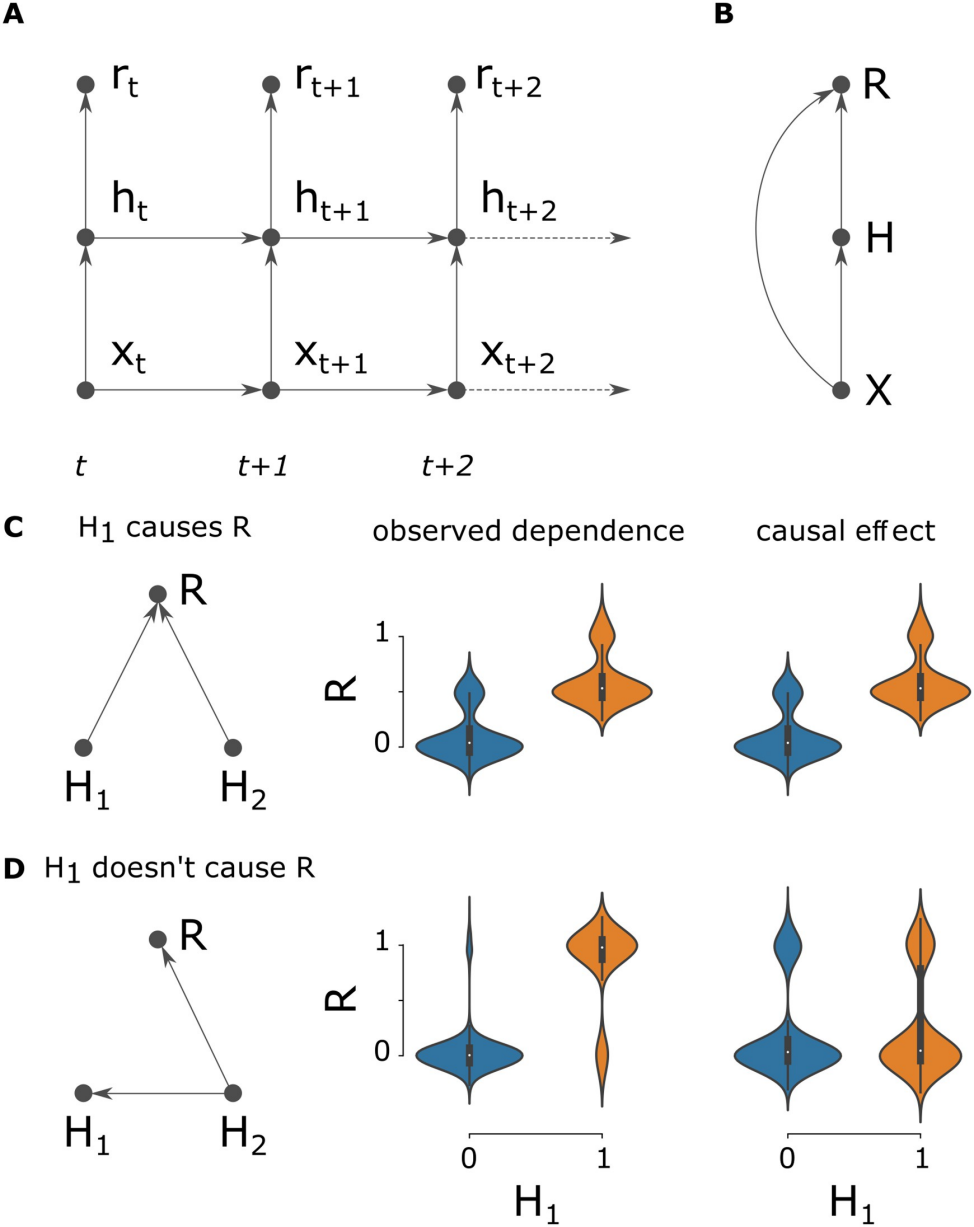
Graphical models formalizing a neuron’s causal effect on reward. (A) The dynamic spiking network model. The state at time bin *t* depends on both the previous state and a hierarchical dependence between inputs **x**_*t*_, neuron activities **h**_*t*_, and the reward signal *r*. Omitted for clarity are the extra variables that determine the network state (*v*(*t*) and *s*(*t*)). (B) To formulate the supervised learning problem, these variables are aggregated in time to produce summary variables of the state of the network during the simulated window. We consider these variables being drawn IID from a distribution (**X**, **Z**, **H**, **S**, *R*) ∼ *ρ*. Intervening on the underlying dynamic variables changes the distribution *ρ* accordingly. E.g. severing the connection from **x**_*t*_ to **h**_*t*_ for all *t* renders **H** independent of **X**. Thus the graphical model over (**X**, **Z**, **H**, *R*) has the same hierarchy (ordering) as the underlying dynamical model. However, the aggregate variables do not fully summarize the state of the network throughout the simulation. Therefore, unlike the structure in the underlying dynamics, **H** may not fully separate **X** from *R*—we must allow for the possibility of a direct connection from **X** to *R*. (C) and (D) are simple examples illustrating the difference between observed dependence and causal effect. We have omitted the dependence on *X* for simplicity. Violin plots show reward when *H*_1_ is active or inactive, without (left subplot) and with (right) intervening on *H*_1_. (C) If *H*_1_ and *H*_2_ are independent, the observed dependence matches the causal effect. (D) If *H*_2_ causes *H*_1_ then *H*_2_ is an unobserved confounder, and the observed dependence and causal effects differ.

To use this theory, first, we describe a graph $G_{\Phi }$ such that *ρ* is compatible with the conditional independence requirement of the above definition. Consider the ordering of the variables Φ that matches the feedforward structure of the underlying dynamic feedforward network (Fig 1A). From this ordering we construct the graph $G_{\Phi }$ over the variables Φ (Fig 1B). This graph respects the order of variables implied in Fig 1A, but it is over-complete, in the sense that it also contains a direct link between *X* and *R*. This direct link between *X* and *R*, though absent in the underlying dynamical model, cannot be ruled out in a distribution over the aggregate variables, so must be included. The graph is directed, acyclic and fully-connected. Being fully connected in this way guarantees that we can factor the distribution *ρ* with the graph $G_{\Phi }$ and it will obey the conditional independence criterion described above.

The second criterion is that the graph can be used to describe what happens when interventions are made. Fully spelling this out is beyond the scope of this study, so here we assume the interventional distributions on nodes of $G_{\Phi }$ factor the distribution *ρ* as expected in the definition above. This is reasonable since, for instance, intervening on the underlying variable *h*_*i*_(*t*) (to enforce a spike at a given time), *would* sever the relation between *Z*_*i*_ and *H*_*i*_ as dictated by the graph topology. Taken together, this means the graph $G_{\Phi }$ describes a causal Bayesian network over the distribution *ρ*.

Given this causal network, we can then define a neuron’s causal effect. In causal inference, the causal effect can be understood as the expected difference in an outcome *R* when a ‘treatment’ *H*_*i*_ is exogenously assigned. The causal effect of neuron *i* on reward *R* is defined as:

$$\beta_{i}≔E\left(R|\text{do}\left(H_{i}=1\right)\right)-E\left(R|\text{do}\left(H_{i}=0\right)\right),$$

where do represents the do-operator, notation for an intervention [27]. Because of the fact that the aggregated variables maintain the same ordering as the underlying dynamic variables, there is a well-defined sense in which *R* is indeed an effect of *H*_*i*_, not the other way around, and therefore that the causal effect *β*_*i*_ is a sensible, and not necessarily zero, quantity (Fig 1C and 1D). That is to say, it makes sense to associate with each neuron, for each stimulus, what its causal effect is on the output and thus reward.

#### Result II: Causal effects and finite difference approximation of gradients

The causal effect *β*_*i*_ is an important quantity for learning: if we know how a neuron contributes to the reward, the neuron can change its behavior to increase it. More specifically, in a spiking neural network, the causal effect can be seen as a type of finite difference approximation of the partial derivative (reward with a spike vs reward without a spike). That is, since we assume that *R* is function of some filtered neural output of the network, then it can be shown that the causal effect, *β*_*i*_, under certain assumptions, does indeed approximate the reward gradient $\frac{\partial R}{\partial S_{i}}$. That is, there is a sense in which:

$$\begin{array}{c} \beta_{i}=E\left(R|\text{do}\left(H_{i}=1\right)\right)-E\left(R|\text{do}\left(H_{i}=0\right)\right)\approx E(\frac{\partial R}{\partial S_{i}}). \end{array}$$ (1)

More specifically:

$$\begin{array}{c} \beta_{i}\approx \Delta_{s}E\left(D_{i}R|\text{do}\left(H_{i}=0\right)\right), \end{array}$$

where *D*_*i*_*R* is a random variable that represents the finite difference operator of *R* with respect to neuron *i*’s firing, and Δ_*s*_ is a constant that depends on the spike kernel *κ* and acts here like a kind of finite step size. Refer to the methods section for the derivation. This result establishes a connection between causal inference and gradient-based learning. It suggests that methods from causal inference may provide efficient algorithms to estimate reward gradients, and thus can be used to optimize reward. In this way the causal effect is a relevant quantity for learning.

### Estimating a neuron’s causal effect using the spiking discontinuity

Having understood how the causal effect of a neuron can be defined, and how it is relevant to learning, we now consider how to estimate it. The key observation of this paper is to note that a discontinuity can be used to estimate causal effects, without randomization, but while retaining the benefits of randomization. We will refer to this approach as the Spiking Discontinuity Estimator (SDE). This estimator is equivalent to the regression discontinuity design (RDD) approach to causal inference which is popular in economics [28, 29].

As outlined in the introduction, the idea is that inputs that place a neuron close to its spiking threshold can be used in an unbiased causal effect estimator. For inputs that place the neuron just below or just above its spiking threshold, the difference in the state of the rest of the network becomes negligible, the only difference is the fact that in one case the neuron spiked and in the other case the neuron did not. Any difference in observed reward can therefore *only* be attributed to the neuron’s activity. Statistically, within a small interval around the threshold spiking becomes *as good as random* [28–30]. In this way the spiking discontinuity may allow neurons to estimate their causal effect.

To demonstrate the idea that a neuron can use its spiking non-linearity to estimate causal effects, here we analyze a simple two neuron network obeying leaky integrate-and-fire (LIF) dynamics. The neurons receive a shared scalar input signal *x*(*t*), with added separate noise inputs *η*_*i*_(*t*), that are correlated with coefficient *c*. Each neuron weighs the noisy input by *w*_*i*_. The correlation in input noise induces a correlation in the output spike trains of the two neurons [31], thereby introducing confounding. At the end of a trial period *T*, the neural output determines a reward signal *R*. Most aspects of causal inference can be investigated in a simple, few-variable model such as this [32], thus demonstrating that a neuron can estimate a causal effect in this simple case is an important first step to understanding how it can do so in a larger network.

To give intuition into how this confounding problem manifests in a neural learning setting, consider how a neuron can estimate its causal effect. A basic model of a neuron’s effect on reward is that it can be estimated from the following piece-wise constant model of the reward function:

$$\begin{array}{c} R=\gamma_{i}+\beta_{i}H_{i}, \end{array}$$ (2)

That is, there is some baseline expected reward, *γ*_*i*_, and a neuron-specific contribution *β*_*i*_, where *H*_*i*_ represents the spiking indicator function for neuron *i* over the trial of period *T*. Then denote by *β*_*i*_ the causal effect of neuron *i* on the resulting reward *R*.

**Fig 2:**
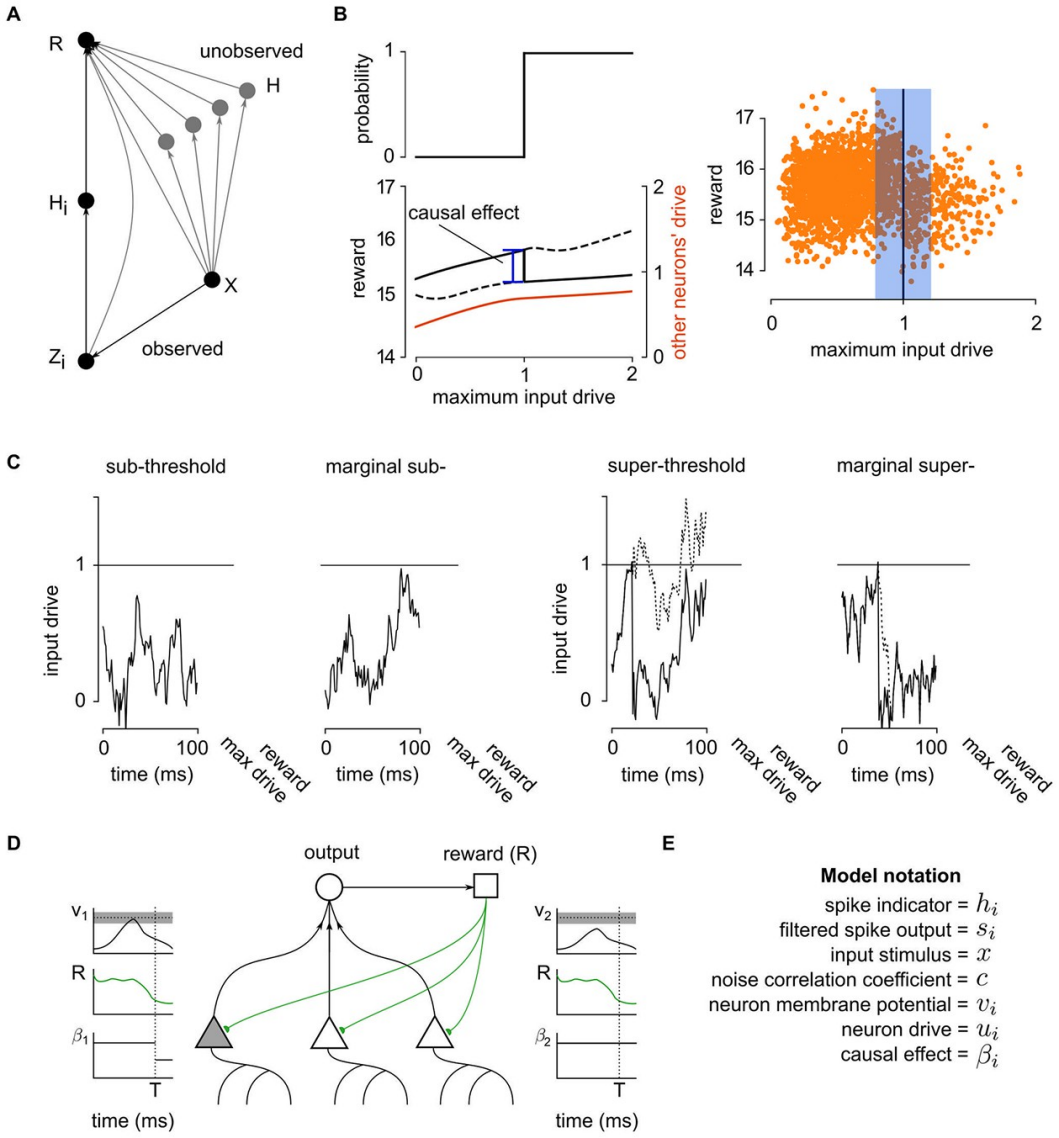
Using the spiking discontinuity to estimate causal effects. (A) Graphical model describing neural network. Neuron *H*_*i*_ receives input **X**, which contributes to drive *Z*_*i*_. If drive is above the spiking threshold, then *H*_*i*_ is active. The activity contributes to reward *R*. Though not shown, this relationship may be mediated through downstream layers of a neural network, and complicated interactions with the environment. From neuron *H*_*i*_’s perspective, the activity of the other neurons **H** which also contribute to *R* is unobserved. For clarity, other neuron’s *Z* variables have been omitted from this graph. Unobserved dependencies of *X* → *R* and *Z*_*i*_ → *R* are shown here, even though not part of the underlying dynamical model, such dependencies in the graphical model may still exist, as discussed in the text. (B) The reward may be tightly correlated with other neurons’ activity, which act as confounders. However any discontinuity in reward at the neuron’s spiking threshold can only be attributed to that neuron. The discontinuity at the threshold is thus a meaningful estimate of the causal effect (left). The effect of a spike on a reward function can be determined by considering data when the neuron is driven to be just above or just below threshold (right). (C) This is judged by looking at the neural drive to the neuron over a short time period. Marginal sub- and super-threshold cases can be distinguished by considering the maximum drive throughout this period. (D) Schematic showing how spiking discontinuity operates in network of neurons. Each neuron contributes to output, and observes a resulting reward signal. Learning takes place at end of windows of length *T*. Only neurons whose input drive brought it close to, or just above, threshold (gray bar in voltage traces; compare neuron 1 to 2) update their estimate of *β*. (E) Model notation.

Estimating *β*_*i*_ naively as

$$\begin{array}{c} \beta_{i}^{OD}=E\left(R|H_{i}=1\right)-E\left(R|H_{i}=0\right), \end{array}$$ (3)

is biased if *H*_*i*_ is correlated with other neuron’s activity (Fig 2A). Call this naive estimator the *observed dependence*.

Instead, we can estimate *β*_*i*_ only for inputs that placed the neuron close to its threshold. That is, let *Z*_*i*_ be the maximum integrated neural drive to the neuron over the trial period. If *θ* is the neuron’s spiking threshold, then a maximum drive above *θ* results in a spike, and below *θ* results in no spike. The neural drive used here is the leaky, integrated input to the neuron, that obeys the same dynamics as the membrane potential except without a reset mechanism. By tracking the maximum drive attained over the trial period, we can track when inputs placed the neuron close to its threshold, and marginally super-threshold inputs can be distinguished from well-above-threshold inputs, as required for SDE (Fig 2C). Let *p* be a window size within which we are going to call the integrated inputs *Z*_*i*_ ‘close’ to threshold, then the SDE estimator of *β*_*i*_ is:

$$\begin{array}{c} \beta_{i}^{SDE}=E\left(R|\theta \leq Z_{i}<\theta +p\right)-E\left(R|\theta -p<Z_{i}<\theta \right). \end{array}$$ (4)

A one-order-higher model of the reward adds a linear correction, resulting in the piece-wise linear model of the reward function:

$$\begin{array}{c} R=\gamma_{i}+\beta_{i}H_{i}+\left[\alpha_{ri}H_{i}+\alpha_{li}\left(1-H_{i}\right)\right]\left(Z_{i}-\theta \right), \end{array}$$ (5)

where *γ*_*i*_, *α*_*li*_ and *α*_*ri*_ are the linear regression parameters. This higher-order model can allow for larger window sizes *p*, and thus a lower variance estimator. Call the causal effect estimate using the piecewise constant model $\beta_{i}^{SDE,0}$ and the causal effect estimate using the piecewise-linear model $\beta_{i}^{SDE,1}$. Both such models are explored in the simulations below.

This approach relies on some assumptions. First, a neuron assumes its effect on the expected reward can be written as a function of *Z*_*i*_ which has a discontinuity at *Z*_*i*_ = *θ*, such that, in the neighborhood of *Z*_*i*_ = *θ*, the function can be approximated by either its 0-degree (piecewise constant version) or 1-degree Taylor expansion (piecewise linear). This approach also assumes that the input variable *Z*_*i*_ is itself a continuous variable. This is the case in simulations explored here.

To summarize the idea: for a neuron to apply spiking discontinuity estimation, it simply must track if it was close to spiking, whether it spiked or not, and observe the reward signal. Then the comparison in reward between time periods when a neuron almost reaches its firing threshold to moments when it just reaches its threshold allows for an unbiased estimate of its own causal effect (Fig 2D and 2E). Below we gain intuition about how the estimator works, and how it differs from the naive estimate.

### A simple empirical demonstration of SDE

**Fig 3:**
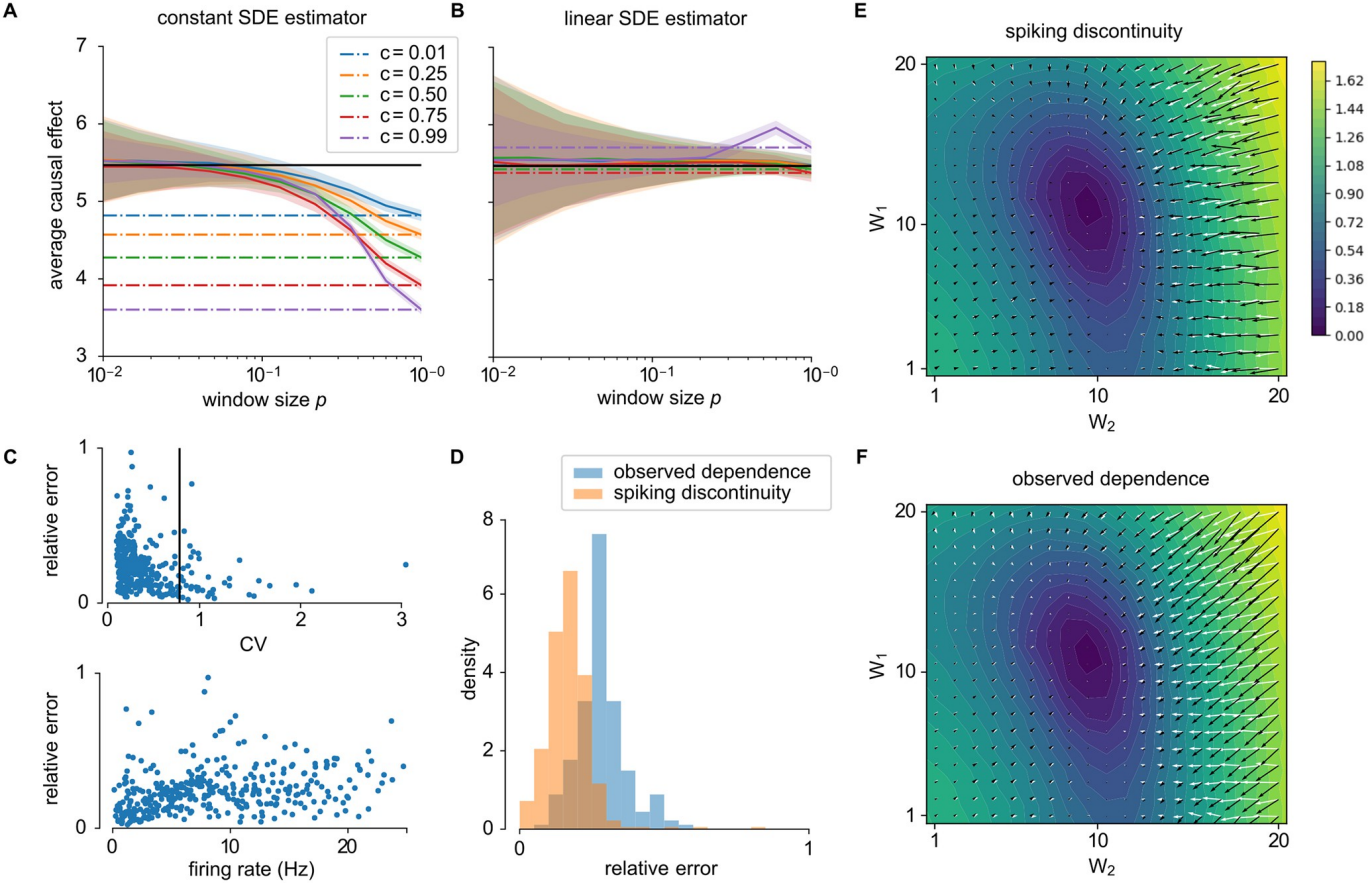
Estimating reward gradient with spiking discontinuity in two-neuron network. (A) Estimates of causal effect (black line) using a constant spiking discontinuity model (difference in mean reward when neuron is within a window *p* of threshold) reveals confounding for high *p* values and highly correlated activity. *p* = 1 represents the observed dependence, revealing the extent of confounding (dashed lines). Curves show mean plus/minus standard deviation over 50 simulations. (B) The linear model is unbiased over larger window sizes and more highly correlated activity (high *c*). (C) Relative error in estimates of causal effect $\beta_{i}^{SDE,0}$ over a range of weights (1 ≤ *w*_*i*_ ≤ 20) show lower error with higher coefficient of variability (CV; top panel), and lower error with lower firing rate (bottom panel). (D) Over this range of weights, spiking discontinuity estimates $\beta_{i}^{SDE,0}$ are less biased than just the naive observed dependence. (E,F) Approximation to the reward gradient overlaid on the expected reward landscape. The white vector field corresponds to the true gradient field, the black field correspond to the spiking discontinuity estimate $\beta_{i}^{SDE,0}$ (E) and observed dependence (F) estimates. The observed dependence is biased by correlations between neuron 1 and 2—changes in reward caused by neuron 1 are also attributed to neuron 2.

Simulating this simple two-neuron network shows how a neuron can estimate its causal effect using the SDE (Fig 3A and 3B). To show how it removes confounding, we implement both the piece-wise constant and piece-wise linear models for a range of window sizes *p*. When *p* is large, the piece-wise constant model corresponds to the biased observed-dependence estimator, $\beta_{i}^{OD}$, while small *p* values approximate the SDE estimator and result in an unbiased estimate (Fig 3A). The window size *p* determines the variance of the estimator, as expected from theory [33]. The piece-wise linear model, Eq (5), is more robust to confounding (Fig 3B), allowing larger *p* values to be used. Thus the linear correction that is the basis of many RDD implementations [28] allows neurons to more readily estimate their causal effect. This linear dependence on the maximal voltage of the neuron may be approximated by plasticity that depends on calcium concentration; such implementation issues are considered in the discussion.

The same simple model can be used to estimate the dependence of the quality of spike discontinuity estimates on network parameters. To investigate the robustness of this estimator, we systematically vary the weights, *w*_*i*_, of the network. This allows for an exploration SDE’s performance in a range of network states. SDE works better when activity is fluctuation-driven and at a lower firing rate (Fig 3C). Thus spiking discontinuity is most applicable in irregular but synchronous activity regimes [26]. Over this range of network weights, spiking discontinuity is less biased than the observed dependence (Fig 3D). Overall, corrected estimates based on spiking considerably improve on the naive implementation.

### Learning with a discontinuity-based causal effect estimator

We just showed that the spiking discontinuity allows neurons to estimate their causal effect. We can implement this as a simple learning rule that illustrates how knowing the causal effect impacts learning. We derive an online learning rule that estimates *β*, and the linear model parameters, if needed. That is, let **u**_*i*_ be the vector of parameters required to estimate *β*_*i*_ for neuron *i*. For the piece-wise constant reward model that is **u**_*i*_ = [*γ*_*i*_, *β*_*i*_] and for the piece-wise linear model it is **u**_*i*_ = [*γ*_*i*_, *β*_*i*_, *α*_*ri*_, *α*_*li*_]. Then the learning rule takes the form:

$$\begin{array}{c} \Delta u_{i}=\{\begin{array}{cc} -\eta \left[u_{i}^{T}a_{i}-R\right]a_{i}, & \theta -p\leq Z_{i}<\theta +p\;\text{(just/almost}\;\text{spikes)};\\ 0, & \text{(otherwise)}, \end{array} \end{array}$$ (6)

where *η* is a learning rate, and **a**_*i*_ are drive-dependent terms (see Methods for details and the derivation). Using this learning rule to update **u**_*i*_, along with the relation

$$\begin{array}{c} \frac{\partial }{\partial w_{i}}E\left(R\right)\approx \frac{\partial E(H_{i})}{\partial w_{i}}\beta_{i}, \end{array}$$ (7)

allows us to update the weights according to a stochastic gradient-like update rule:

$$\Delta w_{i}\propto \frac{\partial }{\partial w_{i}}E\left(R\right),$$

in order to maximize reward. This allows us to use the causal effect to estimate $\frac{\partial R}{\partial w_{i}}$ (Fig 3E and 3F), and thus gives a local learning rule that approximates gradient-descent.

**Fig 4:**
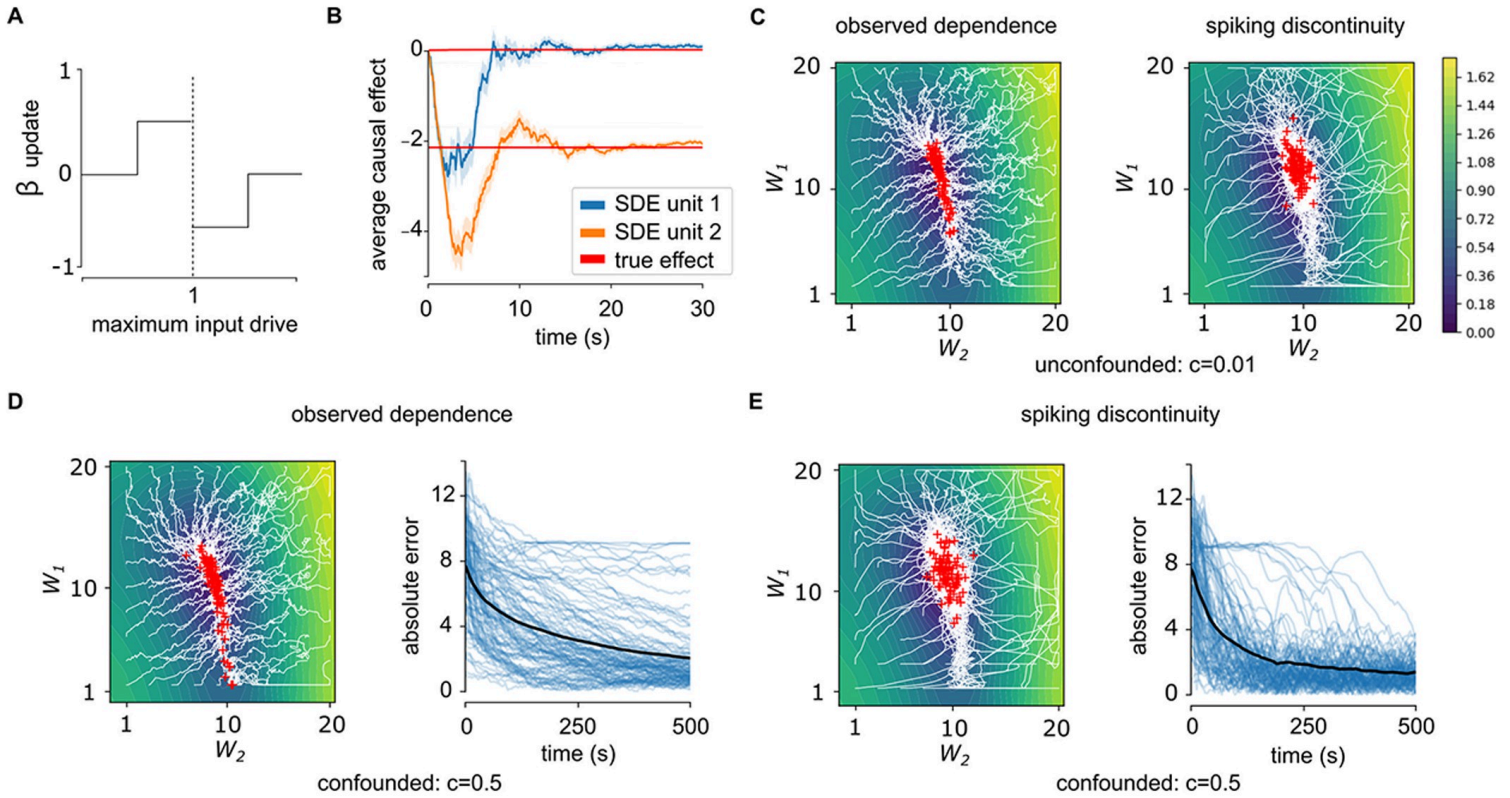
Maximizing reward with a spike-discontinuity learning rule. (A) Parameters for causal effect model, **u**, are updated based on whether neuron is driven marginally below or above threshold. (B) Applying rule to estimate $\beta_{i}^{SDE,0}$ for two sample neurons shows convergence within 10s (red curves). Error bars represent standard error of the mean over 50 simulations. (C) Convergence of observed dependence (left) and spike discontinuity (right) learning rule to unconfounded network (*c* = 0.01). Observed dependence converges more directly to bottom of valley, while spiking discontinuity learning trajectories meander more, as the initial estimate of causal effect takes more inputs to update. (D,E) Convergence of observed dependence (D) and spiking discontinuity (E) learning rule to confounded network (*c* = 0.5). Right panels: error as a function of time for individual traces (blue curves) and mean (black curve). With confounding learning based on observed dependence converges slowly or not at all, whereas spike discontinuity learning succeeds.

When applied to the toy LIF network, the online learning rule (Fig 4A) estimates *β* over the course of seconds (Fig 4B). The estimated *β* is then used to update weights to maximize expected reward in an unconfounded network (uncorrelated noise—*c* = 0.01). In these simulations updates to *β* are made when the neuron is close to threshold, while updates to *w*_*i*_ are made for all time periods of length *T*. Learning exhibits trajectories that initially meander while the estimate of *β* settles down (Fig 4C). When a confounded network (correlated noise—*c* = 0.5) is used the spike discontinuity learning exhibits similar performance, while learning based on the observed dependence sometimes fails to converge due to the bias in gradient estimate. In this case convergence is faster than learning based on observed dependence (Fig 4D and 4E). Thus the spike discontinuity learning rule allows a network to be trained even in the presence of confounded inputs.

### The impact of network depth and width on causal effect estimation

**Fig 5:**
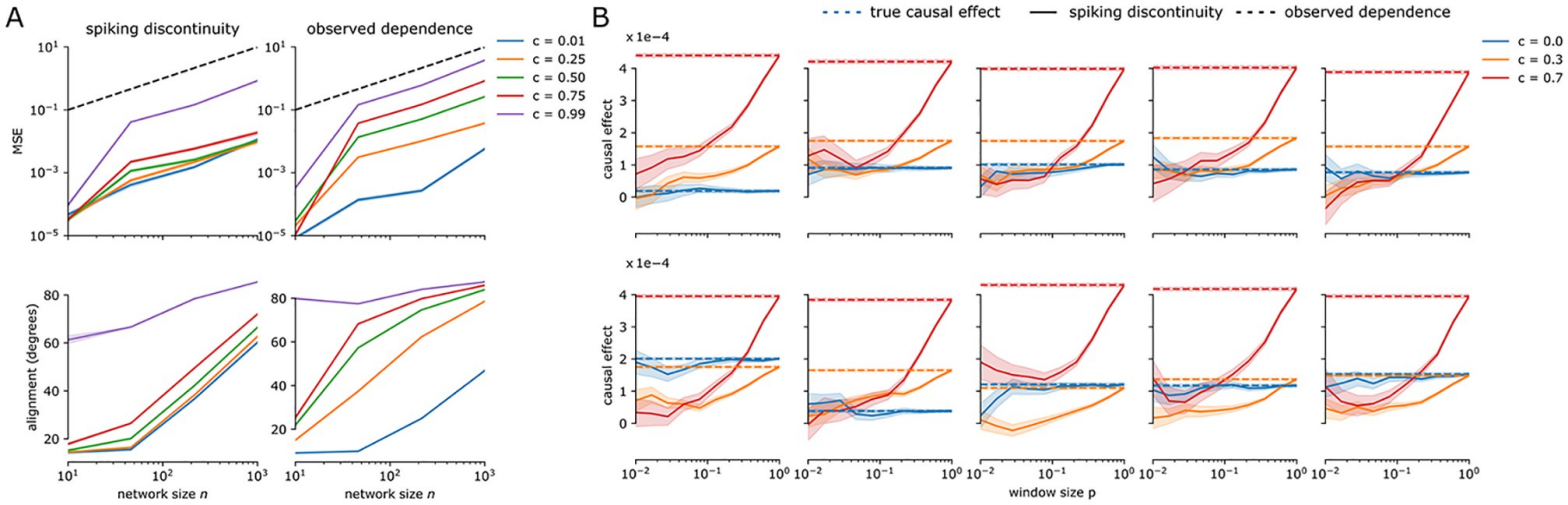
Effect of network architecture on spiking discontinuity. (A) Mean square error (MSE) as a function of network size and noise correlation coefficient, *c*. MSE is computed as squared difference from the true causal effect, where the true causal effect is estimated using the observed dependence estimator with *c* = 0 (unconfounded). Alignment between the true causal effects *β* and the estimated effects ${\hat{\beta }}^{SDE,0}$ is the angle between these two vectors. (B) A two hidden layer neural network, with hidden layers of width 10. Plots show the causal effect of each of the first hidden layer neurons on the reward signal. Dashed lines show the observed-dependence estimator, solid lines show the spiking discontinuity estimator, for correlated and uncorrelated (unconfounded) inputs, over a range of window sizes *p*. The observed dependence estimator is significantly biased with confounded inputs.

Having validated spiking discontinuity-based causal inference in a small network, we investigate how well we can estimate causal effects in wider and deeper networks. First we investigate the effects of network width on performance. We simulate a single hidden layer neural network of varying width (Fig 5A; refer to the Methods for implementation details). The mean squared error in estimating causal effects shows an approximately linear dependence on the number of neurons in the layer, for both the observed-dependence estimator and the spiking discontinuity. For low correlation coefficients, representing low confounding, the observed dependence estimator has a lower error. However, once confounding is introduced, the error increases dramatically, varying over three orders of magnitude as a function of correlation coefficient. In contrast, the spiking discontinuity error is more or less constant as a function of correlation coefficient, except for the most extreme case of *c* = 0.99. This shows that over a range of network sizes and confounding levels, a spiking discontinuity estimator is robust to confounding.

Furthermore, when considering a networks’ estimates as a whole, we can compare the vector of estimated causal effects ${\hat{\beta }}^{SDE,0}$ to the true causal effects *β* (Fig 5A, bottom panels). The angle between these two vectors gives an idea of how a learning algorithm will perform when using these estimates of the causal effect. If considered as a gradient then any angle well below ninety represents a descent direction in the reward landscape, and thus shifting parameters in this direction will lead to improvements. In this case there is a more striking difference between the spiking discontinuity and observed dependence estimators. Only for extremely high correlation values or networks with a layer of one thousand neurons does it fail to produce estimates that are not very well aligned with the true causal effects. In contrast, the observed dependence estimator is only well-aligned with the true gradient for small networks, and with a small correlation coefficient. This suggests the SDE provides a more scale-able and robust estimator of causal effect for the purposes of learning in the presence of confounders.

We also want to know whether spiking discontinuity can estimate causal effects in deep neural networks. It effectively estimates the causal effect in a spiking neural network with two hidden layers (Fig 5B). We compare a network simulated with correlated inputs, and one with uncorrelated inputs. The estimates of causal effect in the uncorrelated case, obtained using the observed dependence estimator, provide an unbiased estimator the true causal effect (blue dashed line). The causal effect in the correlated inputs case is indeed close to this unbiased value. In contrast, using the observed-dependence estimator on the confounded inputs significantly deviates from the true causal effect. These results show spiking discontinuity can estimate causal effects in both wide and deep neural networks.

## Discussion

Here we focused on the relation between gradient-based learning and causal inference. Our approach is inspired by the regression discontinuity design commonly used in econometrics [34]. We cast neural learning explicitly as a causal inference problem, and have shown that neurons can estimate their causal effect using their spiking mechanism. In this way we found that spiking can be an advantage, allowing neurons to quantify their causal effect in an unbiased way.

It is important to note that other neural learning rules also perform causal inference. Thus the spiking discontinuity learning rule can be placed in the context of other neural learning mechanisms. First, as many authors have noted, any reinforcement learning algorithm relies on estimating the effect of an agent’s/neuron’s activity on a reward signal. Learning by operant conditioning relies on learning a causal relationship (compared to classical conditioning, which only relies on learning a correlation) [27, 35–37]. Causal inference is, at least implicitly, the basis of reinforcement learning.

There is a large literature on how reinforcement learning algorithms can be implemented in the brain. It is well known there are many neuromodulators which may represent reward or expected reward, including dopaminergic neurons from the substantia nigra to the ventral striatum representing a reward prediction error [25, 38]. Many of these methods use something like the REINFORCE algorithm [39], a policy gradient method in which locally added noise is correlated with reward and this correlation is used to update weights. This gives an unbiased estimate of the causal effect because the noise is assumed to be independent, private to each neuron. These ideas have extensively been used to model learning in brains [16–22].

Learning in birdsong is a particularly well developed example of this form of learning [17]. In birdsong learning in zebra finches, neurons from area LMAN synapse onto neurons in area RA. These synapses are referred to as ‘empiric’ synapses, and are treated by the neurons as an ‘experimenter’, producing random perturbations which can be used to estimate causal effects. This is a compelling account of learning in birdsong, however it relies on the specific structural form of the learning circuit. It is unknown more broadly how a neuron may estimate what is perturbative noise without these structural specifics, and thus if it can provide an account of learning in general.

There are two factors that cast doubt on the use of reinforcement learning-type algorithms broadly in neural circuits. First, even for a fixed stimulus, noise is correlated across neurons [11–15]. Thus if the noise a neuron uses for learning is correlated with other neurons then it can not know which neuron’s changes in output is responsible for changes in reward. In such a case, the synchronizing presynaptic activity acts as a confounder. Thus, as discussed, such algorithms require biophysical mechanisms to distinguish independent perturbative noise from correlated input signals in presynaptic activity, and in general it is unclear how a neuron can do this. Though well characterized in sensory coding, noise correlation role in learning has been less studied. This work suggests that understanding learning as a causal inference problem can provide insight into the role of noise correlations in learning.

Learning with perturbations scales poorly with network size [17, 22, 40]. Thus neurons may use alternatives to these reinforcement-learning algorithms. A number of authors have looked to learning in artificial neural networks for inspiration. In artificial neural networks, the credit assignment problem is efficiently solved using the backpropagation algorithm, which allows efficiently calculating gradients. Backpropagation requires differentiable systems, which spiking neurons are not. Indeed, cortical networks often have low firing rates in which the stochastic and discontinuous nature of spiking output cannot be neglected [41]. It also requires full knowledge of the system, which is often not the case if parts of the system relate to the outside world. No known structures exist in the brain that could exactly implement backpropagation. Yet, backpropagation is significantly more efficient than perturbation-based methods—it is the only known algorithm able to solve large-scale problems at a human-level [42]. The success of backpropagation suggests that efficient methods for computing gradients are needed for solving large-scale learning problems.

An important disclaimer is that the performance of local update rules like SDE-based learning are likely to share similar scaling to that observed by REINFORCE-based methods, e.g. significantly slower than backpropagation. SDE-based learning, on its own, is not a learning rule that is significantly more efficient than REINFORCE, instead it is a rule that is more robust to the structure of noise that REINFORCE-based methods utilize. Thus it is an approach that can be used in more neural circuits than just those with special circuitry for independent noise perturbations. Such an approach can be built into neural architectures alongside backpropagation-like learning mechanisms, to solve the credit assignment problem. Indeed, this exact approach is taken by [43]. Thus SDE-based learning has relevance to both spiking neural networks in the presence of noise correlations, and as part of a biologically plausible solution to the credit assignment problem.

Further, the insights made here are relevant to models that attempt to mimic backpropagation through time. A lot of recurrent neural networks, when applied to spiking neural networks, have to deal with propagating gradients through the discontinuous spiking function [5, 44–48]. A common strategy is to replace the true derivative of the spiking response function (either zero or undefined), with a pseudo-derivative. A number of replacements have been explored: [44] uses a boxcar function, [47] uses the negative slope of the sigmoid function, [45] explores the so-called straight-through estimator—pretending the spike response function is the identity function, with a gradient of 1. Thus a number of choices are possible. As a supplementary analysis (S1 Text and S3 Fig), we demonstrated that the width of the non-zero component of this pseudo-derivative can be adjusted to account for correlated inputs. We found that in highly correlated cases, learning is more efficient when the window is smaller. Thus the exact same considerations raised by framing learning as a causal inference problem provides insight into other biologically-plausible, spiking learning models.

Finally, it must be noted how the learning rule derived here relates to the dominant spike-based learning paradigm—spike timing dependent plasticity (STDP [49]). STDP performs unsupervised learning, so is not directly related to the type of optimization considered here. Reward-modulated STDP (R-STDP) can be shown to approximate the reinforcement learning policy gradient type algorithms described above [50, 51]. Thus R-STDP can be cast as performing a type of causal inference on a reward signal, and shares the same features and caveats as outlined above. Thus we see that learning rules that aim at maximizing some reward either implicitly or explicitly involve a neuron estimating its causal effect on that reward signal. Explicitly recognizing this can lead to new methods and understanding.

### Compatibility with known physiology

There are a number of ways that the simulations presented here are simplifications of true learning circuits. For instance, the LIF neural network implemented in Figs 2–4 has a fixed threshold. Yet cortical neurons do not have a fixed threshold, but rather one that can adapt to recent inputs [52]. We note that our exploration of learning in this more complicated case—the delayed XOR model (S1 Text)—consists of populations of LIF and adaptive LIF neurons. The adaptive LIF neurons do have a threshold that adapts, based on recent spiking activity. The results in this model exhibit the same behavior as that observed in previous sections—for sufficiently highly correlated activity, performance is better for a narrow spiking discontinuity parameter *p* (cf. Fig 2A). This suggests populations of adaptive spiking threshold neurons show the same behavior as non-adaptive ones. This makes sense since what is important for the spike discontinuity method is that the learning rule use a signal that is unique to an individual neuron. The stochastic, all-or-none spiking response provides this, regardless of the exact value of the threshold. The neuron just needs to know if it was close to its threshold or not. Of course, given our simulations are based on a simplified model, it makes sense to ask what neuro-physiological features may allow spiking discontinuity learning in more realistic learning circuits.

If neurons perform something like spiking discontinuity learning we should expect that they exhibit certain physiological properties. In this section we discuss the concrete demands of such learning and how they relate to past experiments. First, the spiking regime is required to be irregular [26] in order to produce spike trains with randomness in their response for repeated inputs. Irregular spiking regimes are common in cortical networks (e.g. [53]). Further, for spiking discontinuity learning, plasticity should be confined to cases where a neuron’s membrane potential is close to threshold, regardless of spiking. This means inputs that place a neuron close to threshold, but do not elicit a spike, still result in plasticity. This type of sub-threshold dependent plasticity is known to occur [54–56]. This also means that plasticity will not occur for inputs that place a neuron too far below threshold. In fact, in past models and experiments testing voltage-dependent plasticity, changes do not occur when postsynaptic voltages are too low [57, 58]. Spiking discontinuity predicts that plasticity does not occur when postsynaptic voltages are too high. However, in many voltage-dependent plasticity models, potentiation does occur for inputs well-above the spiking threshold. To test if this is an important difference between what is statistically correct and what is more readily implementable in neurophysiology, we experimented with a modification of the learning rule, which does *not* distinguish between barely above threshold inputs and well above threshold inputs. For the piecewise-linear model, this modification did not adversely affect the neurons’ ability to estimate causal effects (S2 Fig). Thus threshold-adjacent plasticity as required for spike discontinuity learning appears to be compatible with neuronal physiology.

The learning rules presented here also need knowledge of outcomes and would thus likely be dependent on neuromodulation. Neuromodulated-STDP is well studied in models [51, 59, 60]. However, this learning requires reward-dependent plasticity that differs depending on if the neuron spiked or not. This may be communicated by neuromodulation. For instance, there is some evidence that the relative balance between adrenergic and M1 muscarinic agonists alters both the sign and magnitude of STDP in layer II/III visual cortical neurons [59]. To the best of our knowledge, how such behavior interacts with postsynaptic voltage dependence as required by spike discontinuity is unknown.

Overall, the structure of the learning rule is that a global reward signal (potentially transmitted through neuromodulators) drives learning of a variable, *β*, inside single neurons. This internal variable is combined with a term $\frac{d}{d\text{w}}E\left(H\right)$ to update synaptic weights. The update rule for the weights depends only on pre- and post-synaptic terms, with the post- term getting updated over time, independently of the weight updates. Such an interpretation is interestingly in line with recently proposed ideas on inter-neuron learning, e.g., Gershman 2023 [61], who proposes an interaction of intra-cellular variables and synaptic learning rules can provide a substrate for memory. Thus, taken together, these factors show that SDE-based learning may well be compatible with known neuronal physiology.

### Conclusion

Here we presented the first exploration of the idea that neurons can perform causal inference using their spiking mechanism. There are a number of avenues for future work to develop the idea further. In particular, we primarily presented empirical results demonstrating the idea in numerous settings. Further experiments with the impact of learning window size in other learning rules where a pseudo-derivative type approach to gradient-based learning is applied can be performed, to establish the broader relevance of the insights made here. And the theoretical results that we presented were made under the strong assumption that the neural network activity, when appropriately aggregated, can be described by a causal Bayesian network. More rigorous results are needed. The problem of coarse-graining, or aggregating, low-level variables to obtain a higher-level model amenable to causal modeling is a topic of active research [62, 63]. Further fleshing out an explicit theory that relates neural network activity to a formal causal model is an important future direction.

In this paper SDE based learning was explored in the context of maximizing a reward function or minimizing a loss function. But the same mechanism can be exploited to learn other signals—for instance, surprise (e.g. [64]) or novelty (e.g. [65]). SDE-based learning is a mechanism that a spiking network can use in many learning scenarios.

The most important aspect of spike discontinuity learning is the explicit focus on causality. A causal model is one that can describe the effects of an agent’s actions on an environment. Learning through the reinforcement of an agent’s actions relies, even if implicitly, on a causal understanding of the environment [37, 66]. Here, by explicitly casting learning as a problem of causal inference we have developed a novel learning rule for spiking neural networks. We present the first model to propose a neuron does causal inference. We believe that focusing on causality is essential when thinking about the brain or, in fact, any system that interacts with the real world.

## Methods

### Neuron simulations and noise

We consider the activity of a simulated network of *n* neurons whose activity is described by their spike times, ${\left\{t_{s}^{i}\right\}}_{s=1}^{M_{i}}$:

$$h_{i}\left(t\right)=\sum_{s=1}^{M_{i}}\delta \left(t-t_{s}^{i}\right).$$

The neurons obey leaky integrate-and-fire (LIF) dynamics

$$\begin{array}{c} {\dot{v}}_{i}=-g_{L}v_{i}+w_{i}\eta_{i}, \end{array}$$ (8)

where integrate and fire means simply:

$$v_{i}\left(t^{+}\right)=v_{r},\;\text{when}\;v_{i}\left(t\right)=\theta .$$

A refractory period of 3ms is added to the neurons. Different choices of refractory period were not shown to affect SDE performance (S1 Fig). Noisy input *η*_*i*_ is comprised of a common DC current, *x*, and noise term, *ξ*(*t*), plus an individual noise term, *ξ*_*i*_(*t*):

$$\eta_{i}\left(t\right)=x+\sigma_{i}[\sqrt{1-c}\xi_{i}\left(t\right)+\sqrt{c}\xi \left(t\right)].$$

The noise processes are independent white noise: $E\left(\xi_{i}\left(t\right)\xi_{j}\left(t^{\prime }\right)\right)=\sigma^{2}\delta_{ij}\delta \left(t-t^{\prime }\right)$. This parameterization is chosen so that the inputs *η*_1,2_ have correlation coefficient *c*. *σ* = 10 was used for the noise magnitude unless otherwise stated. Simulations are performed with a step size of Δ*t* = 1ms. Here the reset potential was set to *v*_*r*_ = 0. The firing rate of a noisy integrate and fire neuron is

$$\begin{array}{c} \mu_{i}={\left[\frac{1}{g_{L}}\int_{0}^{\infty }\frac{1}{u}(\text{exp}\;(-u^{2}+2y_{i}^{th}u)-\text{exp}\;(-u^{2}+2y_{i}^{r}u))\;du\right]}^{-1}, \end{array}$$ (9)

where $y_{i}^{th}=\left(\theta -w_{i}x\right)/\sigma_{i}$ and $y_{i}^{r}=-w_{i}x/\sigma_{i}$, *σ*_*i*_ = *σw*_*i*_ is the input noise standard deviation [21]. This is used in the learning rule derived below.

We define the input drive to the neuron, *u*_*i*_, as the leaky integrated input without a reset mechanism. That is, over each simulated window of length *T*:

$${\dot{u}}_{i}=-g_{L}u_{i}+w_{i}\eta_{i},\;u_{i}\left(0\right)=v_{i}\left(0\right).$$

The SDE method operates when a neuron receives inputs that place it close to its spiking threshold—either nearly spiking or barely spiking—over a given time window. In order to identify these time periods, the method uses the maximum input drive to the neuron:

$$Z_{i}=\underset{0\leq t\leq T}{\text{max}}\;u_{i}\left(t\right).$$

The input drive is used here instead of membrane potential directly because it can distinguish between marginally super-threshold inputs and easily super-threshold inputs, whereas this information is lost in the voltage dynamics once a reset occurs. Here a time period of *T* = 50ms was used. Reward is administered at the end of this period: *R* = *R*(**s**_*T*_).

In our simulations, the dynamics are standard integrate and fire dynamics, $s\in R^{n}$ and are given by

$$\begin{array}{c} \tau_{s}{\dot{s}}_{i}=-s_{i}+h_{i}\left(t\right), \end{array}$$ (10)

for synaptic time scale *τ*_*s*_ = 0.02s. We thus use a standard model for the dynamics of all the neurons.

### Reward model and causal effect estimation

The simulations for Figs 3 and 4 are about standard supervised learning and there an instantaneous reward is given by R(s)∈R. In order to have a more smooth reward signal, *R* is a function of **s** rather than **h**. The reward function used here has the following form:

$$R\left(s_{1},s_{2}\right)={\left(as_{1}+bs_{2}+bs_{2}^{2}+x\right)}^{2}$$

We choose *a* = −30, *b* = 20, *x* = −4.

The true causal effect was estimated from simulations with zero noise correlation and with a large window size—in order to produce the most accurate estimate possible. Determining the causal effect analytically is in general intractable.

### Learning causal effects through the spiking discontinuity

To remove confounding, spiking discontinuity learning considers only the marginal super- and sub-threshold periods of time to estimate *β*. This works because the discontinuity in the neuron’s response induces a detectable difference in outcome for only a negligible difference between sampled populations (sub- and super-threshold periods). The SDE estimates [28]:

$$\beta_{i}^{SDE}≔\underset{x\to \theta^{+}}{\text{lim}}\;E\left(R|Z_{i}=x\right)-\underset{x\to \theta^{-}}{\text{lim}}\;E\left(R|Z_{i}=x\right),$$

for maximum input drive obtained over a short time window, *Z*_*i*_, and spiking threshold, *θ*; thus, *Z*_*i*_ < *θ* means neuron *i* does not spike and *Z*_*i*_ ≥ *θ* means it does.

To estimate $\beta_{i}^{SDE}$, a neuron can estimate a piece-wise linear model of the reward function:

$$R\left(H_{i},Z_{i}\right)=\gamma_{i}+\beta_{i}H_{i}+\left[\alpha_{ri}H_{i}+\alpha_{li}\left(1-H_{i}\right)\right]\left(Z_{i}-\theta \right),$$

locally, when *Z*_*i*_ is within a small window *p* of threshold. Here *γ*_*i*_, *α*_*li*_ and *α*_*ri*_ are nuisance parameters, and *β*_*i*_ is the causal effect of interest. This means we can estimate $\beta_{i}^{SDE}$ from

$$\begin{array}{c} \beta_{i}\approx R(1,\theta )-R(0,\theta ). \end{array}$$

A neuron can learn an estimate of $\beta_{i}^{SDE}$ through a least squares minimization on the model parameters *β*_*i*_, *α*_*li*_, *α*_*ri*_. That is, for time period *n* (of length *T*), if we let $u_{i}={\left[\gamma_{i},\beta_{i},\alpha_{ri},\alpha_{li}\right]}^{T}$ and $a_{i,n}={\left[1,H_{i,n},H_{i,n}\left(Z_{i,n}-\theta \right),\left(1-H_{i,n}\right)\left(Z_{i,n}-\theta \right)\right]}^{T}$, then if the neuron solves the following minimization:

$${\hat{u}}_{i}={\text{argmin}}_{u}\sum_{n:(\theta -p<z_{i,n}<\theta +p)}^{N}{\left[u_{i}^{T}a_{i,n}-R_{n}\right]}^{2},$$

then that allows it to estimate its causal effect.

A neuron can perform stochastic gradient descent on this minimization problem, giving the learning rule:

$$\Delta u_{i}=-\eta \left[u_{i}^{T}a_{i,n}-R_{t}\right]a_{i,n}$$

for learning rate *η* and for all time periods at which *z*_*i*,*n*_ is within *p* of threshold *θ*.

When estimating the simpler, piece-wise constant model for either side of the threshold, the learning rule simplifies to:

$$\begin{array}{c} \Delta \beta_{i}=-\eta \left[\gamma_{i}+\beta_{i}-R_{t}\right],\;\theta \leq z_{i,n}<\theta +p\;\text{(neuron}\;i\;\text{just}\;\text{spikes)}\\ \Delta \gamma_{i}=-\eta [\gamma_{i}-R_{t}]. \end{array}$$

again for all time periods at which *z*_*i*,*n*_ is within *p* of threshold *θ*. This causal inference strategy, established by econometrics, is ultimately what allows neurons to produce unbiased estimates of causal effects.

### The causal effect as a finite-difference operator

As a discrete event, we are interested not necessarily in the local gradient but in the finite difference between spiking and non-spiking. Importantly, this finite-difference approximation is exactly what our estimator gets at. We present a derivation here.

In particular, we show that

$$\beta_{i}=E\left(R|\text{do}\left(H_{i}=1\right)\right)-E\left(R|\text{do}\left(H_{i}=0\right)\right)\approx E(\frac{\partial R}{\partial S_{i}}).$$

To show this we replace $\frac{\partial }{\partial S_{i}}$ with a type of finite difference operator:

$$D_{i}R\left(S_{i},Q_{j\neq i}\right)≔\frac{1}{\Delta_{s}}(E\left(R|S_{i}+\Delta_{s},Q_{j\neq i}\right)-E\left(R|S_{i},Q_{j\neq i}\right)).$$

Here $Q_{j\neq i}\subset X$ is a set of nodes that satisfy the back-door criterion [27] with respect to *H*_*i*_ → *R*. By satisfying the backdoor criterion we can relate the interventional distribution to the observational distribution. When *R* is a deterministic, differentiable function of **S** and Δ_*s*_ → 0 this recovers the reward gradient $\frac{\partial R}{\partial S_{i}}$ and we recover gradient descent-based learning.

To consider the effect of a single spike, note that unit *i* spiking will cause a jump in *S*_*i*_ compared to not spiking (according to synaptic dynamics). If we let Δ_*s*_ equal this jump then it can be shown that $E(D_{i}R)$ is related to the causal effect.

First, assuming the conditional independence of *R* from *H*_*i*_ given *S*_*i*_ and **Q**_*j*≠*i*_:

$$\begin{array}{cc} \beta_{i} & =E(R|\text{do}(H_{i}=1))-E(R|\text{do}(H_{i}=0))\\ \; & =E(E(R|Q_{j\neq i},H_{i}=1)-E(R|Q_{j\neq i},H_{i}=0))\\ \; & =E(E(E(R|S_{i},Q_{j\neq i})|Q_{j\neq i},H_{i}=1)-E(E(R|S_{i},Q_{j\neq i})|Q_{j\neq i},H_{i}=0)). \end{array}$$ (11)

Now if we assume that on average *H*_*i*_ spiking induces a change of Δ_*s*_ in *S*_*i*_ within the same time period, compared with not spiking, then:

$$\begin{array}{c} \rho \left(s_{i}|Q_{j\neq i},H_{i}=1\right)\approx \rho \left(s_{i}-\Delta_{s}|Q_{j\neq i},H_{i}=0\right). \end{array}$$ (12)

**Fig 6:**
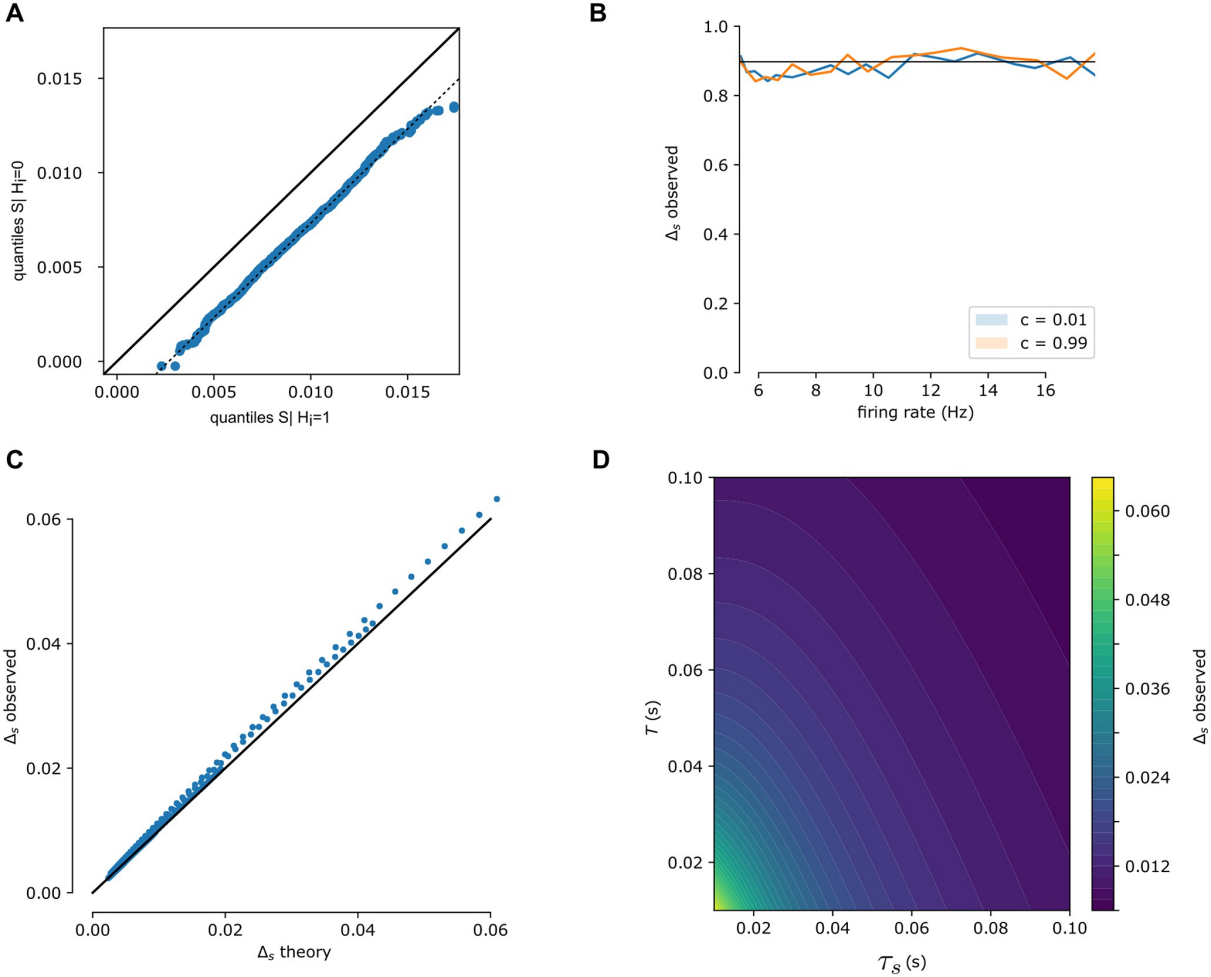
Relation between *S*_*i*_ and *H*_*i*_ over window *T*. (A) Simulated spike trains are used to generate *S*_*i*_|*H*_*i*_ = 0 and *S*_*i*_|*H*_*i*_ = 1. QQ-plot shows that *S*_*i*_ following a spike is distributed as a translation of *S*_*i*_ in windows with no spike, as assumed in (12). (B) This offset, Δ_*s*_, is independent of firing rate and is unaffected by correlated spike trains. (C) Over a range of values (0.01 < *T* < 0.1, 0.01 < *τ*_*s*_ < 0.1) the derived estimate of Δ_*s*_ (Eq (13)) is compared to simulated Δ_*s*_. Proximity to the diagonal line (black curve) shows these match. (D) Δ_*s*_ as a function of window size *T* and synaptic time constant *τ*_*s*_. Larger time windows and longer time constants lower the change in *S*_*i*_ due to a single spike.

Note that *S*_*i*_ given no spike (the distribution on the RHS) is not necessarily zero, even if no spike occurs in that time period, because *s*_*i*_(0), the filtered spiking dynamics at *t* = 0, is not necessarily equal to zero. *s*_*i*_(0) instead has its own, non-zero distribution, representing activity from previous time periods. This approximation is reasonable because the linearity of the synaptic dynamics means that the difference in *S*_*i*_ between spiking and non-spiking windows is simply exp(−(*T* − *t*_*si*_)/*τ*_*s*_)/*τ*_*s*_, for spike time *t*_*si*_. We approximate this term with its mean:

$$\begin{array}{cc} \Delta_{s} & =E(\frac{1}{\tau_{s}}e^{-t_{si}/\tau_{s}}|Q_{j\neq i},H_{i}=1)\\ & \approx \frac{1}{T}(1-e^{-T/\tau_{s}}), \end{array}$$ (13)

under the assumption that spike times occur uniformly throughout the length *T* window. These assumptions are supported numerically (Fig 6).

Writing out the inner two expectations of (11) gives:

$$\begin{array}{cc} & E(E(R|S_{i},Q_{j\neq i})|Q_{j\neq i},H_{i}=1)-E(E(R|S_{i},Q_{j\neq i})|Q_{j\neq i},H_{i}=0)\\ = & \int_{0}^{\infty }E\left(R|Q_{j\neq i},S_{i}=s_{i}\right)[\rho \left(s_{i}|Q_{j\neq i},H_{i}=1\right)-\rho \left(s_{i}|Q_{j\neq i},H_{i}=0\right)]\;ds_{i}\\ \text{from}\;\text{(12)}\;= & \int_{0}^{\infty }E\left(R|S_{i}=s_{i}+\Delta_{s},Q_{j\neq i}\right)\rho \left(s_{i}|Q_{j\neq i},H_{i}=0\right)\\ & -E\left(R|S_{i}=s_{i},Q_{j\neq i}\right)\rho \left(s_{i}|Q_{j\neq i},H_{i}=0\right)\;ds_{i}, \end{array}$$

after making the substitution *s*_*i*_ → *s*_*i*_ + Δ_*s*_ in the first term. Writing this back in terms of expectations gives:

$$\begin{array}{cc} \beta & \approx E(E(E(R|S_{i}+\Delta_{s},Q_{j\neq i})-E(R|S_{i},Q_{j\neq i})|Q_{j\neq i},H_{i}=0))\\ & =\Delta_{s}E(E(D_{i}R\left(S_{i},Q_{j\neq i}\right)\left|Q_{j\neq i},H_{i}=0\right))\\ & =\Delta_{s}E\left(D_{i}R\left(S_{i},Q_{j\neq i}\right)|\text{do}\left(H_{i}=0\right)\right). \end{array}$$

Thus estimating the causal effect is similar to taking a finite difference approximation of the reward.

### Wider and deeper network implementations

We need to give more details for the wide network (Fig 5A) simulations. We use a single hidden layer of *N* neurons each receives inputs *η*. This is a mix of some fixed, deterministic signal and a noise signal, *ξ*:

$$\eta_{i}\left(t\right)=x+\sqrt{1-c}\xi_{i}\left(t\right)+\sqrt{c}\xi \left(t\right).$$

As in the *N* = 2 cases above, this is chosen so that the noise between any pair of neurons is related with a correlation coefficient *c*. This is then weighted by the vector **w**, which drives the leaky integrate and fire neurons to spike. The vector **w** was set to a vector of all ones. The output of the single hidden layer **s** is weighed by a second vector **u**. From this a cost/reward function of *R* = |*U* ⋅ **s** − *Y*| is computed. The vector *U* is randomly generated from a normal distribution N(0,5) such that each neuron has a different causal effect on the output. The target *Y* is set to *Y* = 0.1. From this setup, each neuron can estimate its effect on the function *R* using either the spiking discontinuity learning or the observed dependence estimator. Varying *N* allows us to study how the method scales with network size. Ten simulations were run for each value of *N* and *c* in Fig 5A.

For the deep network (Fig 5B), a two-hidden layer neural network was simulated. Each layer had *N* = 10 neurons. The inputs to the first layer are as above:

$$\eta_{i}^{1}\left(t\right)=x+\sqrt{1-c}\xi_{i}^{1}\left(t\right)+\sqrt{c}\xi^{1}\left(t\right).$$

thus neural activity in this first layer is pairwise correlated with coefficient *c*. The second layer receives input from the first:

$$\eta_{i}^{2}\left(t\right)=V_{i\cdot }s\left(t\right)+\xi_{i}^{2}\left(t\right)$$

thus noise in this layer is not correlated and any correlations between neural activity in the second layer come from correlated activity in the first layer. The matrix *V* weighs inputs from the first to second layer. It is generated from a normal distribution: $N(100,200^{2})$. The same reward function is used as in the wide network simulations above, except here *U* is a vector of ones. The target is set to *t* = 0.02. To test the ability of spiking discontinuity learning to estimate causal effects in deep networks, the learning rule is used to estimate causal effects of neurons in the first layer on the reward function *R*.

## Supporting information

S1 Text Delayed XOR task description, methods and results. (PDF)

S1 Fig Error in causal effect estimation for LIF networks with different refractory periods. As in Fig 3D, histograms plot error in causal effect over a range of network weights. LIF neurons have refractory period of 1,3 or 5 ms. Error is comparable for different refractory periods.
(TIF)

S2 Fig One-sided discontinuity estimator. The one-sided estimator makes updates for inputs placing the neuron *p* below threshold, and for *any* input that places the neuron above threshold. Statistically, the symmetric choice is the most sensible default. Regression discontinuity design, the related method in econometrics, has studied optimizing the underlying kernel, which may not be symmetric depending on the relevant distributions. To address this question, we ran extra simulations in which the window size is asymmetric. We tested, in particular, the case where *p* is some small value on the left of the threshold (sub-threshold inputs), and where *p* is large to the right of the threshold (above-threshold inputs). This deviates from the statistically correct choice, but in a way, this is a biologically plausible setting. This is because, with such a setup, the neuron does not need to distinguish between barely-above-threshold inputs and well-above-threshold inputs, which may be challenging. Instead, any spiking will result in an update to the estimate of the causal effect. We found that the asymmetric estimator performs worse when using the piecewise constant estimator of causal effect, but performs comparably to the symmetric version with using the piecewise linear estimator. Thus spiking discontinuity learning can operate using asymmetric update rules.
(TIF)

S3 Fig Learning delayed XOR in the presence of correlated noise. (A) Delayed XOR task setup, shown after training. Populations of input neurons sequentially encode binary inputs (*x*_1_, *x*_2_), and after a delay a population of neurons cues a response. Input value of 0 is indicated by the red population being active, and 1 is indicated by the blue population being active. Correlated Gaussian noise, with correlation coefficient *c*, is added to the neurons membrane potential. Softmax output above 0.5 indicates a network output of 1, and below 0.5 indicates 0. A sample raster of 20 neurons is shown here. (B) Mean validation error over 10 repeated training runs, for a range of correlation coefficients, *c*, and learning window sizes, *p*. (C) Mean number of iterations taken to reach training error below a stopping threshold of 0.07.
(TIF)
