# Supplementary material for: Neural spiking for causal inference and learning
Source: PLoS Comput Biol. 2023 Apr 4;19(4):e1011005. doi: 10.1371/journal.pcbi.1011005 (PMC10104331; doi:10.1371/journal.pcbi.1011005)
Supplement: S1 Text — (PDF) [file pcbi.1011005.s001.pdf]

Supplementary material

Insights into related learning rules: delayed XOR learning with confounded inputs

Our results show that a neuron's causal effect is measurable in wide and deep networks. How do these insights apply to more challenging learning tasks? Just like other learning rules that in effect perform policy gradient methods to directly optimize to maximize reward (e.g. [1, 2, 3, 4]), the learning rule presented in the main text, though useful to demonstrate the idea, may not scale well to more difficult problems. Instead, the most effective methods for training spiking networks operate by directly trying to implement a form of gradient descent, in effect mimicking the learning algorithms that are successfully used in artificial neural networks. For instance, significant effort has been placed in approximating the back-propagation algorithm in spiking neural networks [5, 6, 7, 8, 9, 10, 11], and many variants of this approach exist. In this section we show how issues of confounding can be successfully mediated by utilizing the spiking nature of neurons in these sorts of models.

A key problem to performing gradient-based optimization in spiking neural networks is how to differentiate through the discontinuous spiking function. In many approaches, to get around the discontinuity of the Heaviside step function, a pseudo-gradient is used. This replaces the derivative of the step function with a continuous alternative. Many such choices are possible [5, 6, 7, 8, 9, 10, 11]. Bellec et al. 2020 [12], for example, uses the following:

$$\frac{dH_i^t}{dv_i^t} = \frac{\gamma}{p\theta} \max\left(0, 1 - \left|\frac{v_i^t - A_i^t}{p\theta}\right|\right),$$

where p is a width parameter, A_i^t is an adaptive firing threshold, for adaptive LIF neurons, and $A_i^t = \theta$ for LIF neurons. Most such choices of pseudo-derivative explored in the literature are centered at the firing threshold, and have some characteristic width beyond which the derivative is zero (p in this example). As a result of this form of approximation, only timesteps in which the neuron is sufficiently close to firing contribute to updating the eligibility. But this is the defining property of the SDE. So, just as in the above examples, it is the case that choosing a sufficiently narrow width can help remove the effects of confounding in the presence of correlated inputs.

We demonstrate this behavior in a delayed XOR learning task. In this task populations of 40 neurons encode two binary variables (x_1, x_2) that are sequentially input to a recurrent, spiking network. After a delay of 250ms, the network is cued to respond with the output $\mathrm{XOR}(x_1, x_2)$ (Fig. S3A). The model setup and parameters are based on Bellec et al. 2020 [12], with the following additions. Gaussian noise is added to the inputs for the neurons, in the same fashion as the above simulations. This noise has correlation coefficient c. The learning window size p is varied to examine performance in different regimes. The model presented in [12] can be considered the special case of $p = 1$. As the use of a pseudo-derivative to perform gradient-based learning in a spiking network is not unique to this method – the insight made here may apply generally to these other approaches too.

A parameter sweep shows that, for low correlations, the network learns faster, and with a smaller validation error, for the largest window sizes – since there are more timesteps in which weights are updated (Fig. S3B,C). For high correlation levels, however, a larger window size is not necessarily better. If the correlation coefficient is too large, though the weights may be updated more frequently, the updates are more likely to be biased by the activity of other neurons which also (almost or barely) fired in that same timestep. For more highly correlated noise, learning is faster and has a lower validation error for intermediate window sizes, around 0.56. This is exactly the behavior that is expected from a confounded learning problem, and that is mitigated through considering the insights of SDE-based learning. Thus the discontinuities utilized in spiking networks can facilitate better learning in the presence of correlated inputs, and this insight is relevant in challenging learning tasks.

References

[1] Xie X, Seung HS. Learning in neural networks by reinforcement of irregular spiking. Physical Review E. 2004;69. doi:10.1016/S0896-6273(03)00761-X.

[2] Fiete IR, Seung HS. Gradient learning in spiking neural networks by dynamic perturbation of conductances. Physical Review Letters. 2006;97. doi:10.1103/PhysRevLett.97.048104.

[3] Fiete IR, Fee MS, Seung HS. Model of Birdsong Learning Based on Gradient Estimation by Dynamic Perturbation of Neural Conductances. Journal of neurophysiology. 2007;98:2038–2057. doi:10.1152/jn.01311.2006.

[4] Seung S. Learning in Spiking Neural Networks by Reinforcement of Stochastics Transmission. Neuron. 2003;40:1063–1073.

[5] Bellec G, Salaj D, Subramoney A, Legenstein R, Maass W. Long short-term memory and Learning-to-learn in networks of spiking neurons. ArXiv e-prints. 2018; p. 1–17.

[6] Huh D, Sejnowski TJ. Gradient Descent for Spiking Neural Networks. Advances in Neural Information Processing Systems. 2017;30.

[7] Neftci EO, Augustine C, Paul S, Detorakis G. Event-driven random back-propagation: Enabling neuromorphic deep learning machines. Frontiers in Neuroscience. 2017;11(JUN):1–18. doi:10.3389/fnins.2017.00324.

[8] Bengio Y, Léonard N, Courville AC. Estimating or Propagating Gradients Through Stochastic Neurons for Conditional Computation. CoRR. 2013;abs/1308.3432.

[9] Shrestha SB, Orchard G. SLAYER: Spike Layer Error Reassignment in Time. In: Proceedings of the 32nd International Conference on Neural Information Processing Systems. NIPS'18. Red Hook, NY, USA: Curran Associates Inc.; 2018. p. 1419–1428.

[10] Zenke F, Ganguli S. SuperSpike: Supervised Learning in Multilayer Spiking Neural Networks. Neural Computation. 2017;30. doi:10.1162/neco_a_01086.

[11] Neftci EO, Mostafa H, Zenke F. Surrogate Gradient Learning in Spiking Neural Networks. IEEE SPM. 2019; p. 1–21.

[12] Bellec G, Scherr F, Subramoney A, Hajek E, Salaj D, Legenstein R, et al. A solution to the learning dilemma for recurrent networks of spiking neurons. Nature Communications. 2020;11(1):1–15. doi:10.1038/s41467-020-17236-y.
