# Supplementary figures and images for: Neural spiking for causal inference and learning
Source: PLoS Comput Biol. 2023 Apr 4;19(4):e1011005. doi: 10.1371/journal.pcbi.1011005 (PMC10104331; doi:10.1371/journal.pcbi.1011005)

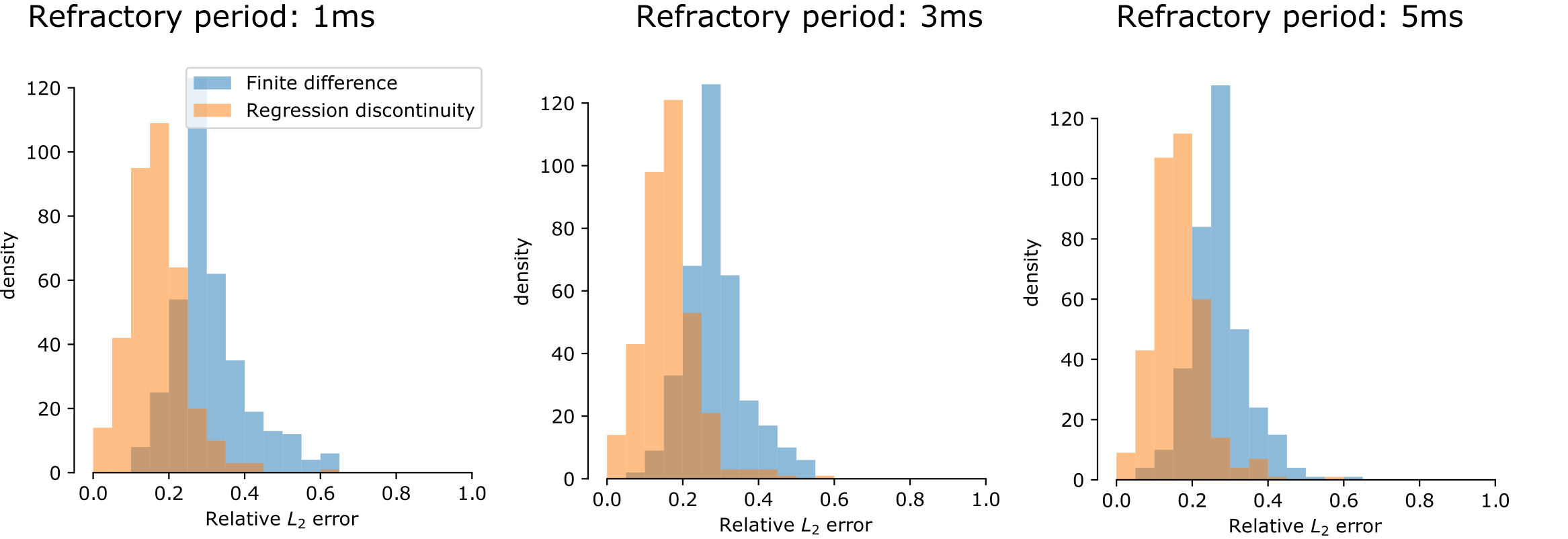

Supplement: S1 Fig — As in Fig 3D, histograms plot error in causal effect over a range of network weights. LIF neurons have refractory period of 1,3 or 5 ms. Error is comparable for different refractory periods. (TIF) [file pcbi.1011005.s002.tif]

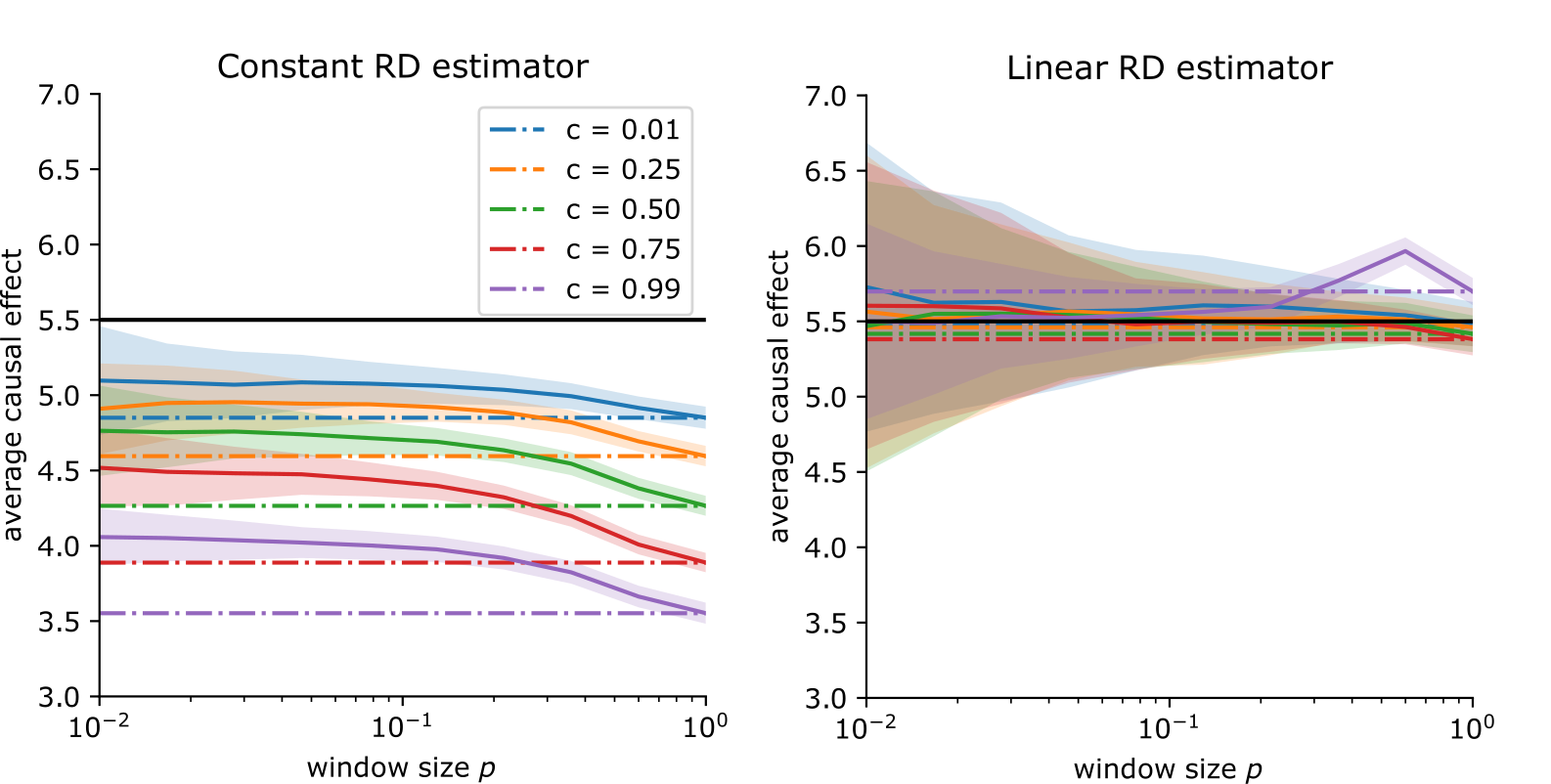

Supplement: S2 Fig — The one-sided estimator makes updates for inputs placing the neuron p below threshold, and for any input that places the neuron above threshold. Statistically, the symmetric choice is the most sensible default. Regression discontinuity design, the related method in econometrics, has studied optimizing the underlying kernel, which may not be symmetric depending on the relevant distributions. To address this question, we ran extra simulations in which the window size is asymmetric. We tested, in particular, the case where p is some small value on the left of the threshold (sub-threshold inputs), and where p is large to the right of the threshold (above-threshold inputs). This deviates from the statistically correct choice, but in a way, this is a biologically plausible setting. This is because, with such a setup, the neuron does not need to distinguish between barely-above-threshold inputs and well-above-threshold inputs, which may be challenging. Instead, any spiking will result in an update to the estimate of the causal effect. We found that the asymmetric estimator performs worse when using the piecewise constant estimator of causal effect, but performs comparably to the symmetric version with using the piecewise linear estimator. Thus spiking discontinuity learning can operate using asymmetric update rules. (TIF) [file pcbi.1011005.s003.tif]

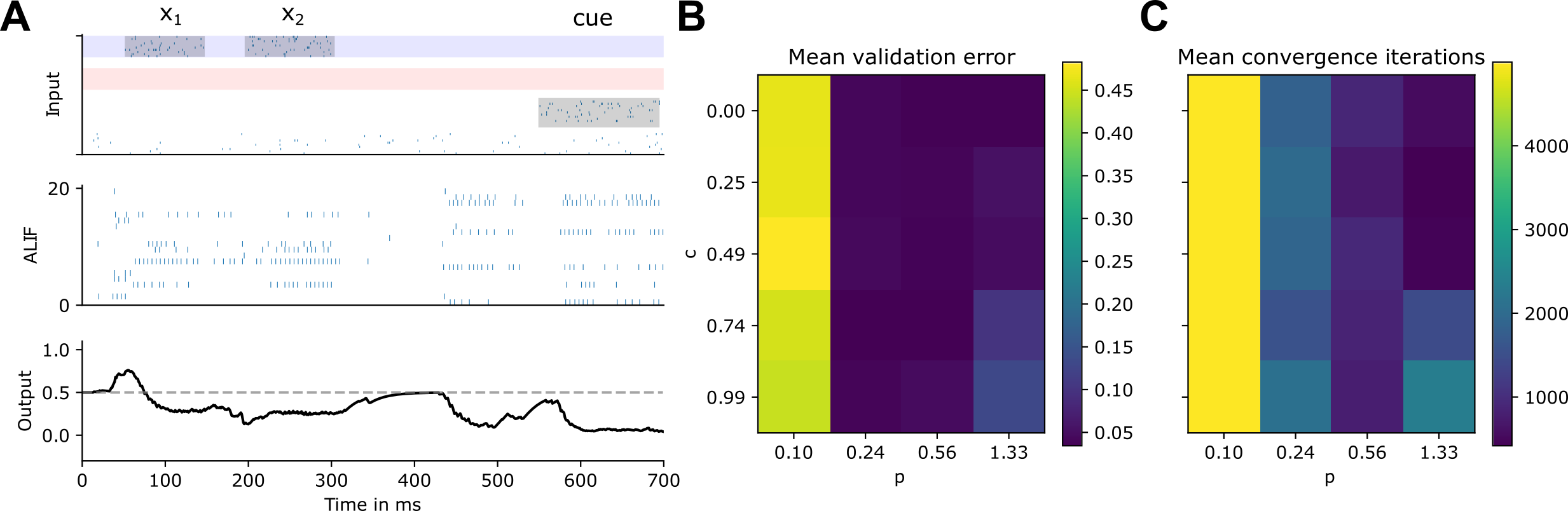

Supplement: S3 Fig — (A) Delayed XOR task setup, shown after training. Populations of input neurons sequentially encode binary inputs (x1, x2), and after a delay a population of neurons cues a response. Input value of 0 is indicated by the red population being active, and 1 is indicated by the blue population being active. Correlated Gaussian noise, with correlation coefficient c, is added to the neurons membrane potential. Softmax output above 0.5 indicates a network output of 1, and below 0.5 indicates 0. A sample raster of 20 neurons is shown here. (B) Mean validation error over 10 repeated training runs, for a range of correlation coefficients, c, and learning window sizes, p. (C) Mean number of iterations taken to reach training error below a stopping threshold of 0.07. (TIF) [file pcbi.1011005.s004.tif]
